# First two years of reimbursed enzyme replacement therapy in the treatment of Fabry disease in Poland

**DOI:** 10.12688/f1000research.55313.1

**Published:** 2021-08-20

**Authors:** Michał Nowicki, Monika Komar, Mariusz Kusztal, Katarzyna Mizia-Stec, Tomasz Liberek, Jolanta Małyszko, Katarzyna Muras-Szwedziak, Krzysztof Pawlaczyk, Piotr Podolec, Jarosław Sławek

**Affiliations:** 1 Department of Nephrology, Hypertension, and Kidney Transplantation, Medical University of Łódź, Łódź, Poland; 2 Department of Cardiac and Vascular Diseases, Institute of Cardiology, Jagiellonian University Medical College, Cracow, Poland; 3 Department of Nephrology and Transplantation Medicine, Wrocław Medical University, Wrocław, Poland; 4 1st Department of Cardiology, Silesian Medical University, Katowice, Poland; 5 Department of Nephrology, Transplantation and Internal Medicine, Medical University of Gdańsk, Gdańsk, Poland; 6 Department of Nephrology, Dialysis and Internal Diseases, Medical University of Warsaw, Warsaw, Poland; 7 Department of Nephrology, Transplantology and Internal Medicine, Poznań University of Medical Sciences Karol Marcinkowski, Poznań, Poland; 8 Department of Neurological-Psychiatric Nursing, Medical University of Gdańsk, Gdańsk, Poland

**Keywords:** Fabry disease, enzyme replacement therapy, ultra-rare disease, α-galactosidase, globotriaosylceramide, globotriaosylsphingosine

## Abstract

Fabry disease (FD) is an ultra-rare genetic lysosomal storage disease caused by pathologic gene variants resulting in insufficient expression of α-galactosidase A. This enzyme deficiency leads to accumulation of globotriaosylceramide and globotriaosylsphingosine in plasma and in different cells throughout the body, causing major cardiovascular, renal, and nervous system complications. Until 2018, reimbursed enzyme replacement therapy (ERT) for FD was available in all European Union countries except Poland.

We present the preliminary results of the first two years of reimbursed ERT in Poland. We obtained data from the seven largest academic centers in Katowice, Kraków, Wrocław, Poznań, Gdańsk, Warszawa, and Łódź. The questionnaire included the following data: number of patients treated, number of patients qualified for ERT, and patient characteristics.

All centers returned completed questionnaires that included data for a total of 71 patients (28 men and 43 women) as of June 2021. Thirty-five patients with the diagnosis of FD confirmed by genetic testing (22 men and 13 women) had already qualified for reimbursed ERT. Mean (SD) age at the commencement of the ERT program was 39.6 (15.5) years (range 18-79 years). Mean time from the first clinical symptoms reported by the patients to the FD diagnosis was 21.1 (8.9) years, and the mean time from the final diagnosis of FD to the beginning of ERT was 4.7 (4.6) years.

FD is still underdiagnosed in Poland. To identify undiagnosed FD patients and to ensure that patients in Poland benefit fully from ERT, implementation of an effective nationwide screening strategy and close cooperation with a network of rare disease centers is advised.

## Introduction

Fabry disease (FD) is an ultra-rare genetic lysosomal storage disease caused by pathologic gene variants resulting in insufficient expression of α-galactosidase A (GLA). ^1^ This enzyme deficiency leads to accumulation of globotriaosylceramide (GL-3) and its deacylated form, globotriaosylsphingosine (lyso-GL-3), in plasma and in different cells throughout the body. Accumulation of GL-3 and lyso-GL-3 causes major organ damage which leads to multiorgan complications involving the central and peripheral nervous systems, kidney, and heart that decrease the quality of life and shorten lifespan. ^1^ FD is largely underdiagnosed and diagnosis is most often established only after target organ damage has already occurred.

The introduction of enzyme replacement therapy (ERT) in 2001 revolutionized the treatment landscape of FD. Clinical studies have proven the efficacy of ERT in the treatment of FD. Specifically, it has been shown that ERT inhibits the progression of target organ damage and stabilizes or even improves organ function. Therefore, international standards and guidelines recommend ERT as the optimal treatment of FD, although for some patients with amenable mutations, an alternative oral chaperone therapy is also available. ^2, 3^

The available ERT treatment currently includes recombinant α-galactosidase A enzymes: agalsidase alfa (Replagal, marketed by Shire) and agalsidase beta (Fabrazyme, marketed by Sanofi Genzyme). In the EU, both agalsidase alfa and agalsidase beta have been approved and available for 20 years. Agalsidase alpha and beta are administered every two weeks by intravenous infusion at a dose of 0.2 mg/kg and 1.0 mg/kg, respectively. ^4, 5^ Although two ERT preparations are currently available and approved for the treatment of FD, there are ongoing debates as to what dose of agalsidase preparation may offer better target organ protection. Two recent national guidelines suggested that higher doses of the recombinant enzyme may result in better clinical outcomes, at least in males with a classic phenotype. ^6, 7^

Until 2018, reimbursed ERT for FD was available in all EU countries except Poland, where only a limited number of patients who participated in clinical trials or compassionate drug use programs received the treatment. ^8^ To help patients with FD obtain access to the therapy, emphasize the challenges they face, and gain public attention, the Association of Families with Fabry Disease, with the help of medical professionals and parliament members, initiated several public campaigns such as “Where is Fabry,” ^9^ “Who is Fabry,” ^10^ and “Fabry Disease – a burning problem”. ^11^ After an initial rejection of the application in 2014, the Polish Ministry of Health eventually included ERT for FD to the list of reimbursable drugs in 2019.

Initially, one of the major challenges in the treatment of FD in Poland was a lack of guidelines for diagnosis and management of the disease. ^12^ In 2020, an interdisciplinary group of Polish clinicians prepared a comprehensive position statement providing practical recommendations for physicians who treat patients with FD. The position statement was approved by the Boards of the Polish Cardiac Society, Polish Society of Inborn Errors of Metabolism, Polish Society of Internal Medicine, Polish Society of Nephrology, and Polish Society of Neurology. ^13^

The introduction of the reimbursable ERT was a major step towards the improvement of the quality of life of Polish patients with FD. However, to date, the results of this treatment program in Poland have not been published. The choice of one of the two available recombinant drugs was the sole decision of the treating physician, but patients had to be centrally approved for the participation in the program by a group of rare disease experts.

## Methods

In 2021, two years after the introduction of reimbursed ERT therapy for FD in Poland, we designed a short survey to gather data on the FD patients currently treated in rare disease centers. The survey was distributed via e-mail to the Fabry disease attending physicians at seven largest academic centers in Katowice, Kraków, Wrocław, Poznań, Gdańsk, Warszawa, and Łódź. The centers were selected based on the number of patients with Fabry disease treated. The questionnaire included the following data: number of patients treated, number of patients qualified for ERT, and patient characteristics (gender, age, date of the qualification to the ERT program, age at the time of qualification, and time from the appearance of the first disease symptoms to the clinical diagnosis).

Data were analyzed using descriptive statistics and Statistica 13.1 PL (StatSoft Polska) software.

## Ethics

The following study was non-interventional, questionnaire-based research, therefore, according to local regulations, Ethics Committee approval and patient informed consent were not required. The authors received permission to collect the data from all the centers involved and the patients' personal data were anonymized.

## Results

All centers returned completed questionnaires that included data for a total of 71 patients (28 men and 43 women) as of June 2021. Thirty-five patients with the diagnosis of FD confirmed by genetic testing (22 men and 13 women) had already qualified for reimbursed ERT. Mean (SD) age at the commencement of the ERT program was 39.6 (15.5) years (range 18-79 years). The mean time from the final diagnosis of FD to the beginning of ERT was 4.7 (4.6) years, although there was a substantial delay from the first clinical symptoms reported by the patients to the diagnosis - 21.1 (8.9) years. The centers with the largest number of patients with FD was Łódź, Cracow, and Wrocław. Detailed numbers of the patients diagnosed and receiving ERT reported by each center in Poland are provided in Table 1.

**Table 1.** Number of patients with Fabry disease treated with enzyme replacement therapy in seven major academic centers in Poland.

Treatment center	Katowice	Poznań	Wrocław	Gdańsk	Kraków	Łódź	Warszawa	Total
**N of treated patients with FD**	3	6	14	2	16	28	2	71
**Men**	2	3	8	2	6	5	2	28
**Women**	1	3	6	0	10	23	0	43
**N of patients treated with ERT**	1	3	6	2	7	12	2	35
**Men**	1	2	6	2	4	5	2	22
**Women**	0	1	2	0	3	7	0	13

ERT, enzyme replacement therapy; FD, Fabry disease.

## Discussion

FD is still underdiagnosed in Poland since the reported disease prevalence and number of patients currently receiving the therapy is lower than in other EU countries. For example, in Germany, the estimated treated FD prevalence was 0.85 per 100,000 insured patients from 2010 to 2017, ^14^ which when extrapolated to the Polish population, may suggest that there should be at least 300 patients with FD that may require specific treatment. The situation is improving since the survey showed that almost half (48%) of the Polish patients with FD are already on reimbursed ERT therapy. This reflects the important role that the program has already played, but much remains to be done to implement an effective nationwide screening strategy to identify undiagnosed FD patients and establish close cooperation with a network of rare disease centers to ensure that patients in Poland benefit fully from ERT.

## Data availability

### Underlying data

Zenodo: First two years of reimbursed enzyme replacement therapy in the treatment of Fabry's disease in Poland. https://doi.org/10.5281/zenodo.5163859. ^15^

This project contains the following underlying data:

- FD Polska Res Letter FINAL database.xlsx.

### Extended data

Zenodo: First two years of reimbursed enzyme replacement therapy in the treatment of Fabry's disease in Poland. https://doi.org/10.5281/zenodo.5163859. ^16^

This project contains the following extended data:

- Copy of survey (translated to English)

Data are available under the terms of the Creative Commons Attribution 4.0 International license (CC-BY 4.0).
