# Supplementary material for: First two years of reimbursed enzyme replacement therapy in the treatment of Fabry disease in Poland
Source: F1000Res. 2021 Oct 22;10:841. Originally published 2021 Aug 20. [Version 2] doi: 10.12688/f1000research.55313.2 (PMC8561609; doi:10.12688/f1000research.55313.2)
Supplement: Peer review history

# Peer review: Reviewer response for version 2

**DOI:** 10.5256/f1000research.78579.r97666

**Published:** 2021-11-01

**Authors:** Bojan Vujkovac

**Affiliations:** 1 Centre for Fabry Disease, General Hospital Slovenj Gradec, Slovenj Gradec, Slovenia

I agree with all answers, comments, and changes the authors made. I have no further comments.

Is the work clearly and accurately presented and does it cite the current literature?

Yes

If applicable, is the statistical analysis and its interpretation appropriate?

Yes

Are all the source data underlying the results available to ensure full reproducibility?

Yes

Is the study design appropriate and is the work technically sound?

Yes

Are the conclusions drawn adequately supported by the results?

Yes

Are sufficient details of methods and analysis provided to allow replication by others?

Yes

Reviewer Expertise:

Fabry Disease, Chronic Kidney Disease

I confirm that I have read this submission and believe that I have an appropriate level of expertise to confirm that it is of an acceptable scientific standard.

# Peer review: Reviewer response for version 2

**DOI:** 10.5256/f1000research.78579.r97665

**Published:** 2021-10-25

**Authors:** Andrzej J. Jaroszynski

**Affiliations:** 1 Department of Nephrology, Collegium Medicum, Jan Kochanowski University of Kielce, Kielce, Poland

The authors addressed all my concerns.

Is the work clearly and accurately presented and does it cite the current literature?

Yes

If applicable, is the statistical analysis and its interpretation appropriate?

Not applicable

Are all the source data underlying the results available to ensure full reproducibility?

Yes

Is the study design appropriate and is the work technically sound?

Yes

Are the conclusions drawn adequately supported by the results?

Yes

Are sufficient details of methods and analysis provided to allow replication by others?

Yes

Reviewer Expertise:

Nephrology, cardiology

I confirm that I have read this submission and believe that I have an appropriate level of expertise to confirm that it is of an acceptable scientific standard.

# Peer review: Reviewer response for version 1

**DOI:** 10.5256/f1000research.58878.r92671

**Published:** 2021-09-13

**Authors:** Bojan Vujkovac

**Affiliations:** 1 Centre for Fabry Disease, General Hospital Slovenj Gradec, Slovenj Gradec, Slovenia

The presenting brief report by Dr. Nowicki and colleagues: "First two years of reimbursed enzyme replacement therapy in the treatment of Fabry disease in Poland" is a very interesting paper showing the importance of available treatment for Fabry disease (FD) patients. According to presenting data, FD patients are managed in the seven largest academic centers in the country. Similar to other countries, also in Poland there is a large time delay from the first clinical sign to the final diagnosis, therefore the authors are correctly pointing out the importance of raising awareness of FD as a rare disease.

 I have just a few minor suggestions for the authors:

**Introduction**

1. (Line 9-10): I suggest emphasizing the importance of early treatment. Namely, disease-specific therapy is efficient only when started before irreversible changes develop. Due to that fact, it is also important to diagnose FD patients at an early age.

**Results:**

1. If possible, it would be of great interest to also include in the Results data on how many families were affected. Namely according to Laney DA *et al.* ^1^, family screening is very effective in diagnosing new patients as there were five family members diagnosed for every proband.
2. Table 1: I would suggest renaming the first group of patients ("N of treated patients with FD" to "N of diagnosed patients with FD)", as it is duplicated and misleading. Also, check the numbers of treated patients with ERT - under Wroclaw numbers and sum are not correct. Check also the final sum of treated in the table and also in text.

**Discussion: **

1. In order to diagnose young patients (i.e. children) and females, the most effective way is family screening. I would suggest adding that fact to the discussion part and explain if it was done or not in Poland. It could be elaborated in a part where you mentioned "effective nationwide screening strategy", which is probably too vague expression and should be explained.
2. Explain the main reasons or obstacles as to why there are still patients not receiving disease-specific treatment despite it being reimbursed.

Is the work clearly and accurately presented and does it cite the current literature?

Yes

If applicable, is the statistical analysis and its interpretation appropriate?

Yes

Are all the source data underlying the results available to ensure full reproducibility?

Yes

Is the study design appropriate and is the work technically sound?

Yes

Are the conclusions drawn adequately supported by the results?

Yes

Are sufficient details of methods and analysis provided to allow replication by others?

Yes

Reviewer Expertise:

Fabry Disease, Chronic Kidney Disease

I confirm that I have read this submission and believe that I have an appropriate level of expertise to confirm that it is of an acceptable scientific standard, however I have significant reservations, as outlined above.

## References

[1] : Diagnosis of Fabry disease via analysis of family history. *J Genet Couns* .2008;17(1) : 10.1007/s10897-007-9128-x 79-83 10.1007/s10897-007-9128-x 18172746

# Response

**Published:** 2021-10-18

**Authors:** Michał Nowicki

**Affiliations:** Department of Nephrology, Hypertension, and Kidney Transplantation, Medical University of Łódź, Poland

Dear Prof. Vujkovac,

 We are very pleased to find out that our study met your interest. We are thankful to the Editor and Reviewers for their comments and suggestions, which undoubtedly improved the quality of our manuscript. We enclose a revised manuscript with introduced changes highlighted as track changes.

 We addressed all the comments as follows:

**Introduction**

- (Line 9-10): I suggest emphasizing the importance of early treatment. Namely, disease-specific therapy is efficient only when started before irreversible changes develop. Due to that fact, it is also important to diagnose FD patients at an early age.

**Author response: **Thank you for raising this issue. We emphasized the need for early treatment introduction at the end of the first paragraph: “Early diagnosis and introduction of disease-specific treatment is essential to stop the disease progression at early stage and prevent from unreversible tissue and organ damage.”.

**Results:**

- If possible, it would be of great interest to also include in the Results data on how many families were affected. Namely according to Laney DA *et al.* ^1^ , family screening is very effective in diagnosing new patients as there were five family members diagnosed for every proband.

**Author response: **Unfortunately, we have no data on family connections of the investigated patients—this issue certainly needs to be explored in further research. We emphasized the need for family screening at the end of the Discussion section as follows: “The screening should particularly concern high-risk groups, i.e., young patients with cardio-vascular accidents and family members of patients already diagnosed with FD.”. We also suggested screening patients with cardiovascular accidents at an early age due to the fact that this is quite a common symptom of FD bringing patients to emergency department units.

- Table 1: I would suggest renaming the first group of patients ("N of treated patients with FD" to "N of diagnosed patients with FD)", as it is duplicated and misleading. Also, check the numbers of treated patients with ERT - under Wroclaw numbers and sum are not correct. Check also the final sum of treated in the table and also in text.

**Author response: **Thank you for pointing this out, we have corrected and double-checked the numbers throughout the table and the manuscript.

**Discussion: **

- In order to diagnose young patients (i.e. children) and females, the most effective way is family screening. I would suggest adding that fact to the discussion part and explain if it was done or not in Poland. It could be elaborated in a part where you mentioned "effective nationwide screening strategy", which is probably too vague expression and should be explained.

**Author response: **We emphasized the need for family screening at the end of the Discussion section as follows: “The screening should particularly concern high-risk groups, i.e., young patients with cardio-vascular accidents and family members of patients already diagnosed with FD.” We also suggested screening patients with cardiovascular accidents at an early age due to the fact that this is quite a common symptom of FD bringing patients to emergency department units.

- Explain the main reasons or obstacles as to why there are still patients not receiving disease-specific treatment despite it being reimbursed.

**Author response: **To our knowledge, the main reason for some patients not receiving disease-specific FD treatment is the reimbursement criteria, which in Poland are particularly strict. Patients qualify to ERT if they are 8 years or older, have FD confirmed in both genetic and enzyme activity testing (however, women with evident clinical symptoms can be qualified despite normal galactosidase activity), and if they present with at least one organ complication, attributed to FD in the differential diagnosis. Unfortunately, this excludes patients with asymptomatic FD, diagnosed during family screening.

# Peer review: Reviewer response for version 1

**DOI:** 10.5256/f1000research.58878.r92673

**Published:** 2021-08-31

**Authors:** Andrzej J. Jaroszynski

**Affiliations:** 1 Department of Nephrology, Collegium Medicum, Jan Kochanowski University of Kielce, Kielce, Poland

This is a well-written manuscript that reports on the situation of FD patients in Poland two years after the introduction of the ERT reimbursement which enabled the treatment of FD patients in this country. The findings are interesting with some novel data presented regarding the relation between the FD diagnosis and the reimbursement of the ERT. However, the authors should address the following issues:

1. Has there been an increase in the number of diagnoses of FD after the introduction of ERT reimbursement? The lack of reimbursement and hence the lack of therapy may be at least partly the reason why the number of FD diagnosed patients per million of the population is much lower in Poland than in other European countries;
2. The authors should also explain why in the eastern part of Poland patients with FD are not treated/diagnosed. I suggest that these two issues should be discussed in the discussion section.

Is the work clearly and accurately presented and does it cite the current literature?

Yes

If applicable, is the statistical analysis and its interpretation appropriate?

Not applicable

Are all the source data underlying the results available to ensure full reproducibility?

Yes

Is the study design appropriate and is the work technically sound?

Yes

Are the conclusions drawn adequately supported by the results?

Yes

Are sufficient details of methods and analysis provided to allow replication by others?

Yes

Reviewer Expertise:

Nephrology, cardiology

I confirm that I have read this submission and believe that I have an appropriate level of expertise to confirm that it is of an acceptable scientific standard, however I have significant reservations, as outlined above.

# Response

**Published:** 2021-10-18

**Authors:** Michał Nowicki

**Affiliations:** Department of Nephrology, Hypertension, and Kidney Transplantation, Medical University of Łódź, Poland

Dear Prof. Jaroszyński,

 We are very pleased to find out that our study met your interest. We are thankful to the Editor and Reviewers for their comments and suggestions, which undoubtedly improved the quality of our manuscript. We enclose a revised manuscript with introduced changes highlighted as track changes.

 We addressed all the comments as follows:

- Has there been an increase in the number of diagnoses of FD after the introduction of ERT reimbursement? The lack of reimbursement and hence the lack of therapy may be at least partly the reason why the number of FD diagnosed patients per million of the population is much lower in Poland than in other European countries;

**Author response: **Thank you for raising this issue. Previous lack of ERT reimbursement indeed seems to be the main cause of the lower number of patients diagnosed with FD compared to other European countries. Unfortunately, we are unable to source the information regarding the number of patients diagnosed with FD before the reimbursement was introduced. We updated the Discussion section accordingly.

- The Authors should also explain why in the eastern part of Poland patients with FD are not treated/diagnosed. I suggest that these two issues should be discussed in the discussion section.

**Author response: **Indeed, our study suggests some disproportions between eastern and western Poland. This might be due to national fund criteria for medical centers that can be contracted for ERT, with only highly specialized facilities being eligible, therefore, some patients need to move to another province to receive treatment. Also, eastern Poland is less populated than the western part. On the other hand, our study concerned only the seven biggest centers, selected based on the number of patients with FD treated. Therefore, in eastern Poland, there may be patients with FD diagnosed and treated, however, the study inclusion criteria did not capture them. We updated the Discussion section accordingly.
